# The Braxton Hicks Method of Treating Placenta Prævia

**Published:** 1886-05-20

**Authors:** 


### THE BRAXTON HICKS METHOD OF TREATING PLACENTA PRÆVIA.

The wonderful statistics of results obtained by the Braxton Hicks method of treating placenta plævia, collected and published by Dr. Lomer, of Berlin, in the *American Journal of Obstetrics*, for December, 1884, have attracted a great deal of notice among obstetricians. So remarkable are they, that published as they were in a special journal, we make no excuse for calling renewed attention to them.

The method, as practiced by its originator, and adopted and described by Dr. Lomer, consists simply in performing the bimanual version, and bringing down a leg. After this the labor is allowed to pursue its own natural course, only helped by gentle traction, if there be too much delay. The points on which Lomer insists are, first, that the version should be at the *very earliest* moment possible, as soon as one or two fingers can be passed through the cervix. It is claimed that in this way the cotton tampon, an uncertain, dangerous (promoting sepsis) and painful appliance. is done away with, and a more certain and effectual tampon brought to bear.

The second point insisted on is that after turning the uterus should be left to expel the child with little or no outside aid, thus giving the os plenty of time to dilate, and avoiding lasceration of the cervix, an accident particularly dangerous under these circumstances.

The matter of bimanual turning does not seem to be sufficiently understood and practiced among us, and yet Lusk declares it to be "one of the most important contributions to obstetric practice of the present century." The method of its performance is very simple. One hand is introduced wholly within the vagina and one or two fingers through the os. These fingers act on the lower segment of the fœtal ovoid, while the hand outside acts in an opposite direction on the other end. In this way the child can be readily and easily turned, especially when the liquor amnii is still present and the uterus relaxed. One reason for failure, when this occurs, we apprehend to be an omission to anæsthetize the patient. This is of the utmost importance, as complete relaxation of the abdominal walls and of the uterus is thus secured and the operation greatly facilitated. It will be at once seen that this method is particularly applicable to the earlier stages of labor, and that the placenta being *prævia* is no insurmountable obstacle. Lomer advises that one edge should be sought and the amniotic sac entered on one side, or failing to find an edge, that the fingers should be passed directly through the placenta itself, a matter of no great difficulty.

Now as to results: They are simply astonishing. Of cases treated by this method alone L. has been able to collect as follows: Hofmeir 37, with 1 death; Behm 40, no deaths; and his own, taken from Schroder's clinic, 101 with 7 deaths; total 178, with a mortality of 8 (4.5 per cent). It may be objected that these were hospital cases under the care of experts with a large experience in this particular operation. B.'s and H.'s cases are open to this objection, but L.'s cases were under the care of nine different operators, many of them beginners. If we take the cases treated by Lomer personally (16), and add them to those already reported, we have a total of 95 with the almost incredible mortality of *one*. But take the worst results of all, the mortality (7 per cent.) in the cases collected by L. (101), and compare it with the mortality generally admitted in the text-books, from 25 to 40 per cent.. and certainly there is a vast difference in favor of the new method, and one which should make it the duty of every one intending to practice obstetrics to familiarize himself with this resource.

Now, as to the method, is it really difficult? Does it need an expert? Lomer assures us that it is not difficult, and the condition under which his results were obtained would seem to bear out his statement. With an experience of two cases, both successful, treated in this way, and a still larger experience of the bimanual version in other classes of cases, we can confidently confirm his statement. Even more, we can say that it is astonishingly easy, easier than the old method by internal version. In our last case, the time elapsing between the introduction of the hand into the vagina and the withdrawal of a foot was certainly not more than two or three minutes, though it was a central implantation and a head presentation. One finger only was passed through the placenta.

All the conditions, if the woman be chloroformed, for a favorable performance of the operation are present; no retraction of the uterus, and most of the liquor amnii present.

But what of the child? Are its chances better or worse? At first sight they might seem to be worse; but Lomer shows, by a large number of statistics, that they are not materially worse than under the old method. In his 101 cases, fifty per cent. were saved, a rather better showing than is made by the majority of the older operators. If the placenta is pierced of course the child is lost; but if a leg can be brought down beside it, and the delay is not too great, its chances are quite as good, or even better, than by tampon internal version and rapid delivery.—*Med. Press.*

